# Commentary: Origin and evolution of pathogenic coronaviruses

**DOI:** 10.3389/fimmu.2020.00811

**Published:** 2020-04-21

**Authors:** Shun Adachi, Takaaki Koma, Naoya Doi, Masako Nomaguchi, Akio Adachi

**Affiliations:** ^1^Department of Microbiology, Kansai Medical University, Hirakata, Japan; ^2^Department of Microbiology, Tokushima University Graduate School of Medical Science, Tokushima, Japan

**Keywords:** COVID-19, pathogenic coronaviruses, SARS-CoV, MERS-CoV, SARS-CoV-2, origin, evolution

One of major study concerns in virology is viral adaptive evolution due to its highly replicable and mutable nature in changeable environments. Some viruses with this property are severely pathogenic for animals and also for humans. Virologists thus must prepare the ground for clinical applications of their findings obtained by structural and functional analyses on viruses. Among viruses, some coronaviruses (CoVs) are notorious for causing the severe acute respiratory syndrome (SARS) and Middle East respiratory syndrome (MERS). Markedly, the very now COVID-19 outbreak is brought about by a new coronavirus designated SARS-CoV-2 (1–3). In this commentary, we focus on the titled review article and mainly introduce evolutionary aspects of the coronaviruses. The said article has successfully predicted today's COVID-19 outbreak by pointing out that novel pathogenic variants will readily emerge from very diversified severe acute respiratory syndrome-related coronaviruses (SARSr-CoVs) of the bat origin through their close coexistence and high genetic recombination ability (Figure 1). Therefore, it is very appropriate and timely to introduce this excellent review article here in the General Commentary article.

**Figure 1:**
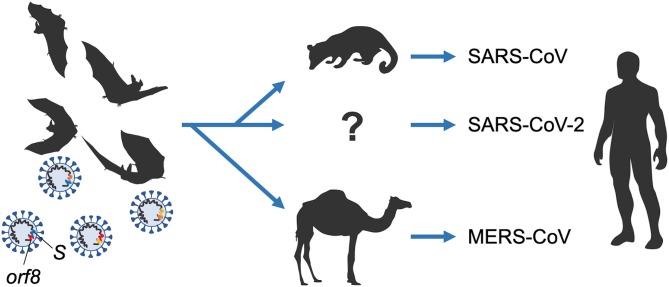
Emergence of coronaviruses pathogenic for humans from ancestral bat viruses. Diversification of SARS-CoV, SARS-CoV-2, and MERS-CoV from ancestor viruses by possible genome recombinations is illustrated. Two major variable regions among viral genomes (*S* and *orf8* genes) are indicated. The variations are most probably due to the high recombination potentials of the coronaviruses. For details, refer to the titled review article in this commentary.

As basal knowledge for coronaviruses, they form an envelope structure at the outer surface of virions and their morphology is spherical with 100–160 nm in diameter. Their genome is positive-sense (+) single-stranded (ss) RNA and 27–32 kb in size. Coronaviruses have extra accessory genes in addition to those for viral structural proteins. Following entry into cells via the specific interaction of viral envelope glycoprotein spike (S) and cellular receptor, coronaviruses replicate in the cytoplasm as the other ssRNA (+) viruses. For alpha- and beta-coronaviruses, they are originated in highly metabolized mammals, such as bats and rodents. After being spilled over to alpacas, cows, civets, camels, or pigs, they can also infect humans and frequently cause SARS and MERS. Gamma- and delta-coronaviruses mainly infect birds, but they sometimes infect mammals. Molecular phylogenetic trees well support this idea of classifications.

Among several genes on the genome of interest, an evolutionary biologist tends to focus on a functionally conserved but sequentially diverged gene. This is because the role for the critical gene is conserved in various species, while it is rapidly evolving, indicating that a diverging force acts on the gene, e.g., by co-evolution such as symbiosis, evolutionary arms race, or others. Of the CoV genes, this review has centered on structural gene *S* and extra genes *orf3*/*orf8* that encode S and accessory proteins, respectively. Importantly, the most frequently observed hotspots for recombination are within *S* gene and upstream region of *orf8* gene. *Orf8* of SARS-CoV is assumed to be acquired from SARSr-CoV by recombination, and is positively selected (4). S protein contains the receptor-binding domain (RBD) critical for infection, and ORF3/ORF8 proteins function viral species-specifically, e.g., by prescribing the virulence (ORF8), anti-interferon activity (ORF3/ORF8) or others. Because ORF3/ORF8 are different between SARS-CoV and SARS-CoV-2, they might contribute to the difference in their virulence (5). Additionally, *S* and *orf3* genes are positively selected in civet SARSr-CoVs (4). Since RNA viruses are easy to mutate and coronaviruses have high potentials for recombination, we can easily see the track of mutations and evolutions of the viruses, especially for SARS-CoV and MERS-CoV. RNA recombination by RNA-dependent RNA polymerase with a low fidelity is widely observed and is supposed to shape current viruses by rearranging their genomes or disseminating functional modules (6). For coronaviruses such as mouse hepatitis virus (MHV), secondary structures of RNA genome are responsible for highly dynamic spike structures (7). These might be an evolutionary cradle from “RNA world,” still function in the living organisms nowadays. The non-processive replicase-driven template switching mechanism proposed among coronaviruses (8) thus is a suitable model for the evolution of RNA-based replicating system. Among many viruses, coronaviruses (recombination frequency for the total genome *in vivo* is 25%) and picornaviruses are champions of the RNA recombination frequency (8).

Let us move to the evolution of coronaviruses (Figure 1). In detail for each gene of the viruses noticed, *orf8* is well-known for viral evolution and the accompanied increase of virulence observed during the late onset of SARS. For the RBD in S, SARS-CoV utilizes ACE2 as a cellular receptor for infection, and MERS-CoV utilizes DPP4 as the receptor. The receptor recognition is important for infection process for the viruses. Different use of the receptors among the coronaviruses is due to their sequences/structures of RBD. Nonetheless, because of the common nature of RBD (9), it can be a promising target for development of novel antiviral compounds and antibody therapies for these viruses. However, it is also true in some cases, viruses went beyond the arms race and ingeniously evolved to counteract the clinical strategy. For example, the strategy is applicable for SARS-CoV WIV1 strain but not for SHC014 and HKU3. HKU3 is remarkable for its truncated form of RBD. For MERS-CoV, cells expressing suboptimal bat species-derived variant of DPP4 force the viruses to accumulate mutations in the viral spike during passages, resulting in enhanced viral entry merely with two amino acid mutations (10). The type of adaptation phenomena in virus evolution is testable either in clinical medicine or *in vitro* evolution system.

Thus, to consider the origin of new pathogens and the prevention of their transmission to humans, and control of the viruses, not only studies on SARS-CoV, MERS-CoV, and SARS-CoV-2, but also those on their relatives SARSr-CoVs and MERSr-CoVs are recommendable for bats tracked for the ecology and evolution. We better understand the interaction networks among viruses of the evolution and diversification by their detailed comparison (Figure 1). The review mentions Yunnan SARSr-CoVs might be the origins of the SARS-CoVs, as symbionts, while domestication activity for mammals affects acquisition of pathogenicity to humans [refer also to Banerjee et al. (11)]. Both fieldworks and experimental biology are required to understand the viruses concomitantly with predicting or preventing potential outbreaks.

## Author Contributions

SA and AA conceived the idea. SA wrote the manuscript. TK, ND, MN, and AA reviewed the manuscript. TK depicted Figure 1. All authors approved its submission.

## Conflict of Interest

The authors declare that the research was conducted in the absence of any commercial or financial relationships that could be construed as a potential conflict of interest.
